# MicroRNA‐199b Modulates Vascular Cell Fate During iPS Cell Differentiation by Targeting the Notch Ligand Jagged1 and Enhancing VEGF Signaling

**DOI:** 10.1002/stem.1930

**Published:** 2015-04-23

**Authors:** Ting Chen, Andriana Margariti, Sophia Kelaini, Amy Cochrane, Shaunta T. Guha, Yanhua Hu, Alan W. Stitt, Li Zhang, Qingbo Xu

**Affiliations:** ^1^ Department of CardiologyThe First Affiliated Hospital, School of Medicine, Zhejiang UniversityHangzhouPeople's Republic of China; ^2^ Centre for Experimental Medicine, School of Medicine, Dentistry and Biomedical Sciences, Queen's University BelfastBelfastUnited Kingdom; ^3^ Cardiovascular DivisionKing's College LondonLondonUnited Kingdom

**Keywords:** Induced pluripotent stem cells, Endothelial cell differentiation, Angiogenesis, Tissue regeneration, Signal transduction

## Abstract

**Aims:** Recent ability to derive endothelial cells (ECs) from induced pluripotent stem (iPS) cells holds a great therapeutic potential for personalized medicine and stem cell therapy. We aimed that better understanding of the complex molecular signals that are evoked during iPS cell differentiation toward ECs may allow specific targeting of their activities to enhance cell differentiation and promote tissue regeneration. **Methods and Results:** In this study, we have generated mouse iPS cells from fibroblasts using established protocol. When iPS cells were cultivated on type IV mouse collagen‐coated dishes in differentiation medium, cell differentiation toward vascular lineages were observed. To study the molecular mechanisms of iPS cell differentiation, we found that miR‐199b is involved in EC differentiation. A step‐wise increase in expression of miR‐199 was detected during EC differentiation. Notably, miR‐199b targeted the Notch ligand JAG1, resulting in vascular endothelial growth factor (VEGF) transcriptional activation and secretion through the transcription factor STAT3. Upon shRNA‐mediated knockdown of the Notch ligand JAG1, the regulatory effect of miR‐199b was ablated and there was robust induction of STAT3 and VEGF during EC differentiation. Knockdown of JAG1 also inhibited miR‐199b‐mediated inhibition of iPS cell differentiation toward smooth muscle markers. Using the in vitro tube formation assay and implanted Matrigel plugs, in vivo, miR‐199b also regulated VEGF expression and angiogenesis. **Conclusions:** This study indicates a novel role for miR‐199b as a regulator of the phenotypic switch during vascular cell differentiation derived from iPS cells by regulating critical signaling angiogenic responses. Stem Cells *2015;33:1405–1418*

## Introduction

Induced pluripotent stem (iPS) cells are able to differentiate into nearly all types of cells 1, 2, 3. This unique characteristic offers significant potential for cell‐replacement therapies to repair tissues or organs damaged by injury, degenerative diseases, aging, or cancer 4, 5, 6. Moreover, the generation of patient‐specific iPS cells offers a promising strategy for autologous cell therapy and personalized medicine 7, 8, 9. iPS cells have been extensively used for generation of endothelial cells (ECs) for in vitro and in vivo studies 2, 10, 11. iPS cells can serve as a very useful tool in establishing efficient protocols of differentiation and developing the platforms to investigate their underlying mechanisms. Since stem cells need to respond to a multitude of extrinsic signals to initiate differentiation, they are intricately connected with their surroundings and receive constant input from their niches, which direct their subsequent behavior 12, 13. Therefore, better understanding of the cellular players and molecular signals that constitute stem cell niches under different physiological and pathological conditions will facilitate development of more refined models of stem cell responses. Subsequently, this may ultimately allow specific targeting of their activities to promote tissue regeneration. For instance it is speculated that if information regarding chromatin states with global views of noncoding RNAs and their regulatory signaling are combined, it is possible to obtain an integrative picture of the molecular mechanisms that govern cell fate specification.

The molecular switches and regulatory pathways that control iPS differentiation to ECs are complex. For example, a number of studies have demonstrated the critical role of microRNAs (miRNAs) in regulating EC and smooth muscle marker (SMC) function 14, 15, 16, 17, 18. miRNAs are a novel class of short (∼22 nucleotides) noncoding RNAs, which are important post‐transcriptional inhibitors of gene expression via base pairing with the three untranslated regions (UTRs) of mRNAs and promoting mRNA instability 19. Evidence has emerged about the crucial involvement of specific miRNAs in the differentiation of pluripotent stem cells into different lineages. One of the miRNAs that have been shown to be implicated in myocardial disease is, for example, miR‐92a (a component of the highly expressed in human ECs “miR‐17approximately92” cluster), which controls new blood vessel formation 8. In the context of vascular disease, the miRNA family containing miR‐199a‐1, miR‐199a‐2, and miR‐199b are potentially important. Encoded by the intron antisense strand of a dynamin gene (Dnm2, Dnm3, and Dnm1, respectively) 9 these miRNAs have been implicated in a number of disease processes 13. Importantly, miR‐199b is modulated in cardiomyocytes and ECs by exposure to hypoxia and high glucose in vitro 20. To date, several studies have demonstrated the regulation of miRNAs in a myriad of vascular biology events. Nevertheless, there is still insufficient evidence to demonstrate how they control EC fate commitment and the mechanisms involved in these differentiation processes.

In this study, we provide evidence that miR‐199b is implicated in EC differentiation derived from iPS cells by modulating vascular endothelial growth factor (VEGF) expression under transcriptional control of STAT3. To shed light on the underlying mechanisms of this regulation in iPS cell‐derived ECs, we have shown that miR‐199b regulates STAT3‐VEGF by targeting the Notch ligand JAG1. Notably, miR‐199b also controls iPS cell differentiation toward SMCs through targeting of JAG1. We therefore propose a novel role for miR‐199b as a potential regulator of the phenotypic switch during vascular cell differentiation.

## Materials and Methods

### Materials

Cell culture media, serum, and cell culture supplements were purchased from ATCC; Manassas, VA, USA, Millipore; USA, Invitrogen; NY, USA, and PAA; North America. Antibodies against VE‐cadherin (ab33168), Flk‐1 (ab9530), VEGFA (ab51745), STAT3 (ab119351), JAG‐1 (ab7771), NOTCH‐1 (ab27526), SMA; Smooth Muscle Actin (ab5831), and SM22 (ab14106) were purchased from Abcam; Cambridge, UK. The Antibody against GAPDH (sc‐25778) was purchased from Santa Cruz. Antibodies against CD31 were purchased from ABBIOTEC; San Diego, USA (250590) and Santa Cruz; Heidelberg, Germany (sc‐1506). The secondary antibodies for immunostaining anti‐mouse Alexa 488 and anti‐rabbit Alexa 488 were purchased from Invitrogen. The secondary antibodies for Western blotting were purchased from DakoCytomation; Glostrup, Denmark and Abcam. Recombinant mouse VEGF was purchased from R and D Systems; Abingdon, UK.

### iPS Cell Culture and Differentiation

Mouse iPS cells were generated in our laboratory as previously described 21. Mouse iPS cells were cultured on gelatin‐coated flasks (phosphate‐buffered saline [PBS] containing 0.04% of gelatin from bovine skin; Sigma) in Dulbecco's modified Eagle's medium (ATCC) supplemented with 10% fetal bovine serum, 100 IU/ml penicillin, and 100 µg/ml streptomycin (Invitrogen); 10 ng/ml recombinant human leukemia inhibitory factor (Millipore); and 0.1 mM 2‐mercaptoethanol (Invitrogen) in a humidified incubator supplemented with 5% CO_2_. The cells were passaged every 2 days at a ratio of 1:6. Differentiation of iPS cells was induced by seeding the cells on type IV mouse collagen (5 µg/ml)‐coated dishes in differentiation medium (DM) that contains α‐MEM supplemented with 10% fetal bovine serum (FBS) (Invitrogen), 0.05 mM 2‐mercaptoethanol, 100 units/ml penicillin, and 100 µg/ml streptomycin in the presence of with 50 ng/ml VEGF from 0 to 8 days.

### Jag1 Substrate

Mouse iPS cells were seeded on 5 µg/ml Jag‐1‐coated plates and cultured in DM media supplemented with VEGF for 6 days. Then the cells were harvested and subjected to further analysis and the expression of EC markers were detected by real‐time PCR. The Recombinant Rat Jagged 1 FC Chimera, (Product Code: 599‐JG100) was purchased from R&D Systems and prepared according to the manufacturer's instructions.

### miR‐199b Transient Transfection

Manipulation of miR‐199b levels in iPS cells differentiated in the presence of VEGF for 4 days and cultured to 60%–70% confluence was performed using a synthetic precursor for miR‐199b (Pre‐199b), or the nontargeting control (Pre‐ctrl) (Ambion AB), whereas inhibition of miR‐199b was performed using the locked nucleic acid (LNA) inhibitor of miR‐199b (LNA‐199b) or a negative control (LNA‐ctrl) (Exiqon). All transfections have been performed using Lipofectamine RNAiMAX (Invitrogen), at a final concentration of 50 nM, according to the manufacturer's protocol. All these experiments have been performed in the presence of the VEGF from day 0 to days 6–7, where the transfected cells were harvested and subjected to further analysis. In order to highlight the important role of miR‐199b in the secretion of the VEGF and EC differentiation, few experiments have been designed and performed in the absence of VEGF. All the experimental conditions have been clearly described in the methods, the results, and the figure legends. To detect miRNAs, total RNA was isolated using the miRVana miRNA isolation kit (Ambion) according to the protocol of the manufacturers. Total and miRNA‐specific cDNA was generated with TaqMan MicroRNA Reverse Transcription Kit (Ambion), and miRVana quantitative reverse transcriptase‐polymerase chain reaction (RT‐PCR) primer sets for miRNAs (Ambion). The PCR reaction was directly monitored by the ABI PRISM 7000 Sequence Detection System (Applied Biosystems, CA). Briefly, cDNA was made from enriched miRNA using the TaqMan MicroRNA RT Kit. U6 RNA was also used as an endogenous control.

### Reverse Transcriptase‐Polymerase Chain Reaction

RT‐PCR and real‐time PCR were performed as described previously 22. The detailed method is shown in Supporting Information Methods S1.

### Quantitative RT‐PCR

Relative gene expression was determined by quantitative real‐time PCR, using 2 ng of cDNA (relative to RNA amount) for each sample with the SYBR Green Master Mix in a 20‐µl reaction. *Ct* values were measured using the ABI Prism 7000 sequence detector (Applied Biosystems). The 18 S ribosomal RNA served as the endogenous control to normalize the amounts of RNA in each sample. For each sample, PCR was performed in duplicate in a 96‐well reaction plate (Eppendorf, twin.tec real‐time PCR plates). The gene was considered undetectable beyond 35 cycles. A primer list is given in Supporting Information Methods S1.

### Immunofluorescence Staining

The procedure used for immunofluorescent staining was similar to that described previously 22. Briefly, cells were fixed with 4% paraformaldehyde and permeabilized with 0.1% Triton X‐100 in PBS for 10 minutes and blocked in 5% swine serum in PBS for 30 minutes at 37°C. The cells were incubated with primary antibody: mouse VEGFR (Flk‐1) or rabbit CD144 for 1 hour at 37°C. The bound primary antibody was revealed by incubation with the secondary antibody; anti‐mouse Alexa488, or anti‐rabbit Alexa488 at 37°C for 30 minutes. Cells were counterstained with 4′,6‐diamidino‐2‐phenylindole (DAPI; Sigma), mounted in Floromount‐G (Cytomation; DAKO, Glostrup, Denmark), and examined with a fluorescence microscope (Axioplan 2 imaging; Zeiss) or SP5 confocal microscope (Leica, Germany).

### Immunoblotting

The method used was similar to that described previously 22. The detailed method is present in Supporting Information Methods S1.

### Lentiviral Particle Transduction

Lentiviral particles were produced using the MISSION shSTAT3, shJAG‐1 DNA plasmids (SIGMA) according to protocol provided and previously described 22. The shRNA Nontargeting vector was used as a negative control. For lentiviral infection, iPS were differentiated for 3 days, and the cells were incubated with shSTAT3, or shJAG‐1 or Nontargeting control (1 × 10^7^ TU/ml) (24 hours prior the transfection with mir‐199b or inhibitor), in complete medium supplemented with 10 µg/ml of Polybrene for 24 hours. Subsequently, fresh medium was added to the cells and the plates were returned to the incubator and harvested 72 hours later for further analysis. The detailed method is shown in Supporting Information Methods S1.

### Luciferase Reporter Assay

For the luciferase reporter assays, 3 × 10^4^ iPS cells were seeded on collagen IV‐coated well of a 12‐well plate in DM containing VEGF. Seventy‐two hours later, cells were transfected with the luciferase plasmids under the control of the promoter of the VEGF receptor (Addgene [plasmid 21307] generated by Mammoto et al.) 23, the JAG1 3′UTR Lenti‐reporter‐Luc Vector (ABM), and the Pre‐199b, LNA‐199b and controls. Briefly, 0.33 µg/well of the reporter plasmids was cotransfected with the Pre‐199b, or LNA‐199b and controls (2 µl/well) using jetPRIME (Polyplus‐transfection SA) according to the protocol provided. pGL3‐Luc Renilla (0.1 µg/well) was included in all transfection assay as internal control. Luciferase and Renilla (Promega) activity assays were detected 48 hours after transfection using a standard protocol 24. The relative luciferase unit was defined as the ratio of luciferase activity to Renilla activity with that of control set as 1.0.

### Enzyme‐Linked Immunosorbent Assay

The concentration of the VEGF released in the supernatant was detected by VEGF ELISA kit (R&D) according to the manufacturers' procedure. Differentiation of iPS cells was induced by seeding the cells on type IV mouse collagen‐coated dishes in DM media supplemented with VEGF. On day 4th, the cells were transfected with Pre‐199b or LNA‐199b and the relative controls (Pre‐Ctrl, LNA‐Ctrl), and 48 hours later, the supernatant have been collected and the secretion of VEGF was quantified by enzyme‐linked immunosorbent assay (ELISA) according to the manufacturer's instructions.

### Fluorescence‐Activated Cell Sorting Analysis

iPS cells were seeded on type IV mouse collagen‐coated dishes in DM media supplemented with VEGF. On day 4th, the cells were transfected with Pre‐199b or LNA‐199b and the relative controls (Pre‐Ctrl, LNA‐Ctrl). Forty‐eight hours later, the cells were harvested and subjected to fluorescence‐activated cell sorting (FACS) analysis for CD31‐FITC (ab13466), rabbit anti‐IgG‐FITC, CD144‐FITC (ab33321, Abcam), SMA (Cy3 [C6198], SIGMA), and IgG‐Cy3 (C2181, Sigma) using a similar protocol as it has been previously described 24. Where an intracellular staining was performed such as intracellular antigen for SMA, the cells were incubated with cold permeabilization buffer (PBS‐0.1% Triton X‐100) for 15 minutes on ice, before the nonspecific antibody binding blocking and primary antibody incubation steps. Data analysis was carried out using Attune Cytometric Software.

### In Vitro Tube Formation Assay

iPS cells were seeded on collagen IV‐coated plates and cultured in DM media in the absence of VEGF for 4 days, then the Pre‐199b or Pre‐Ctrl was introduced to the cells by transfection (in the absence of VEGF). Forty‐eight hours later, the cells have been subjected to Matrigel plugs assays in vitro. Additional experiments were performed in the presence of the VEGF to induce EC differentiation first and then the cells were transfected with LNA‐199b or LNA‐Ctrl. Forty‐eight hours later, the cells have been subjected to Matrigel plugs assays in vitro. In vitro angiogenesis assays were performed after 48 hours as described previously 21, 24. Cell suspension containing 4 × 10^4^ transfected cells was placed on top of the 50 µl/well Matrigel (10 mg/ml; BD Matrigel Basement Membrane Matrix, A6661) in eight‐well chamber slides (Nunc). Rearrangement of cells and formation of capillary‐like structures were observed at 6–12 hours.

### In Vivo Angiogenesis Assay

iPS cells were differentiated and transfected as described above for in in vitro tube formation assays. Forty‐eight hours later, the cells were labeled with Molecular Probes Vybrant Cell Labeling (MP22885) before the in vivo angiogenesis assay in order to distinguish the in vitro‐differentiated cells from the host cells. In vivo angiogenesis was performed by mixing 5 × 10^5^ cells with 200 µl of Matrigel and injecting it subcutaneously in mice (C57BL/6). Six injections were conducted for each group. Seven days later, the mice were killed and the plugs were harvested, frozen in liquid nitrogen, and cryosectioned. Samples were fixed with 4% paraformaldehyde in PBS at 4°C overnight, and then HE staining was performed at room temperature, and were processed with Photoshop software (Adobe). Images were assessed with Axioplan 2 imaging microscope with Plan‐NEOFLUAR ×10, NA 0.3, objective lenses, AxioCam camera, and Axiovision software (all Carl Zeiss MicroImaging, Inc.) All procedures were performed according to protocols approved by the Institutional Committee for Use and Care of Laboratory Animals. All animals used in this study were inbred mice of C57BL/6 background.

### Immunofluorescence Staining and Frozen Section Staining

The procedure for frozen section staining is similar to that described elsewhere 25. In short, Matrigel plugs were harvested, serial 5‐mm‐thick frozen sections were cut from cryopreserved tissue blocks, fixed in a cold 1:1 acetone 10 minutes, and washed with PBS for 20 minutes. After washing with PBS, specimens were placed in a humidified chamber and blocked in 5% swine serum in PBS for 30 minutes at 37°C and incubated with primary antibodies rabbit anti‐VE‐cadherin and rabbit anti‐CD31 and proceed as described in the immunostaining section above. The bound primary antibodies were revealed by incubation with the secondary antibody; anti‐rabbit Alexa488, at 37°C for 30 minutes. Specimens were counterstained with DAPI (Sigma), mounted in Floromount‐G (Cytomation; DAKO), and EC markers, DAPI and Vybrant were examined with a fluorescence microscope (Axioplan 2 imaging; Zeiss) or SP5 confocal microscope (Leica, Germany). Immunostaining was assessed and capillary density was calculated as the number of capillary number/mm^2^.

### Statistical Analysis

Data expressed as the mean ± SEM were analyzed using GraphPad Prism 5 software with a two‐tailed Student's *t* test for two groups or pairwise comparisons. A value of *p* < .05 was considered significant; significance was depicted by asterisks, *, *p* < .05.

## Results

### MiR‐199b Is Implicated in EC Differentiation Derived from iPS Cells

**Figure 1:**
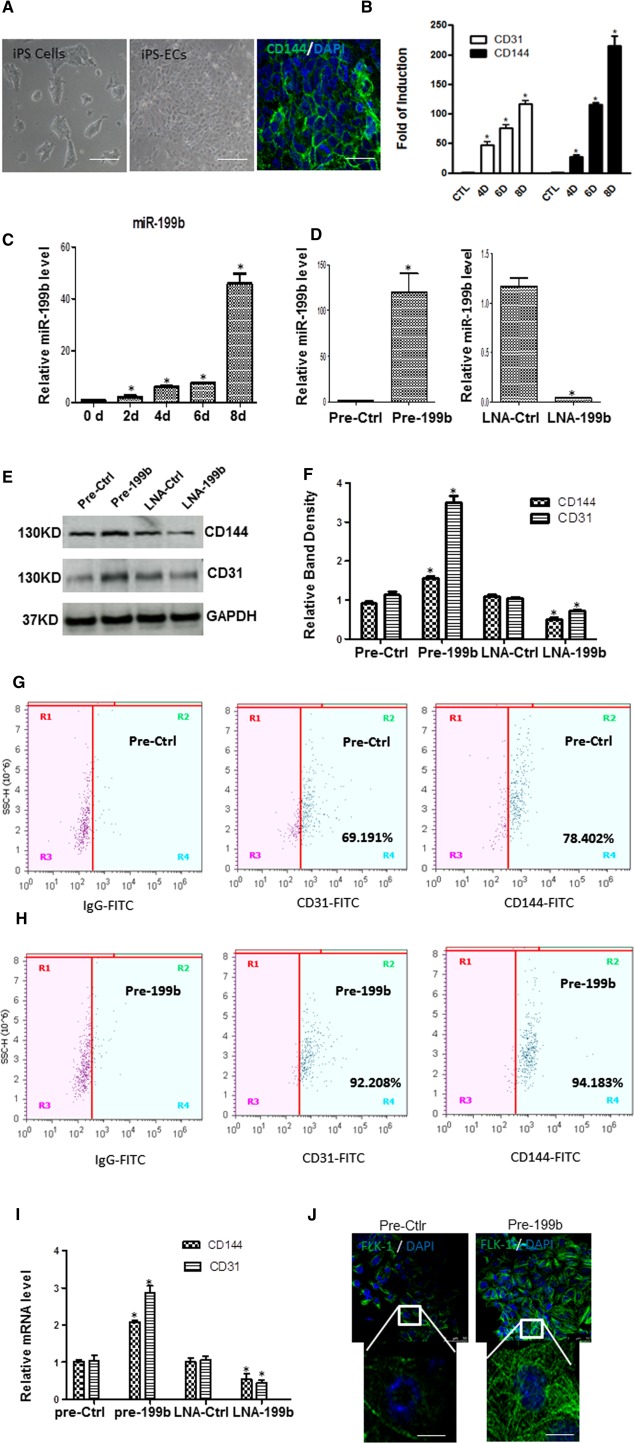
MiR‐199b is implicated in EC differentiation derived from iPS cells. **(A):** Mouse iPS cells were seeded on collagen IV‐coated dishes and cultured in differentiated media supplemented with 50 ng/ml vascular endothelial growth factor (VEGF). Images show the morphology of undifferentiated iPS cells (left panel), differentiated into ECs (iPS‐ECs) on day 8 (middle panel), and immunofluorescent images of CD144‐positive iPS‐ECs on day 8 during EC differentiation. DAPI was used and stained the cell nucleus. Scale bar = 50 µm. **(B):** The endothelial markers CD31 and CD144 are induced in a time point manner during EC differentiation from iPS cells in the mRNA level as real‐time PCR experiments demonstrated (data are means ± SEM; *n* = 3), *, *p* < .05. **(C):** The expression of miR‐199b was found to be upregulated during EC differentiation in a time point experiment as real‐time PCR revealed (data are means ± SEM; *n* = 3), *, *p* < .05. **(D):** iPS cells were forced to differentiate toward ECs for 4 days by seeding the cells on collagen IV in the presence of differentiation medium supplemented with VEGF and then efficiently transfected with either Pre‐199b precursor molecules (Pre‐199b) or an miR‐199b inhibitor (LNA‐199b) resulted in significant Pre‐199b upregulation and miR‐199b repression, respectively (D). On day 7, the cells were harvested and further analyzed, revealing that Pre‐199b increased the expression of endothelial markers CD144 and CD31 in both protein (**E, F** [quantification]) and mRNA levels as it is shown by real‐time PCR **(I)**. LNA‐199b decreased the EC marker expression in protein (E, F) and mRNA levels (I) (data are means ± SEM; *n* = 3), *, *p* < .05. **(G, H):** Fluorescence‐activated cell sorting analysis was performed and confirmed that Pre‐199b induced the expression of EC markers CD31, and CD144 during EC differentiation. **(J):** Immunofluorescence images showed that Pre‐199b induced the expression of VEGF receptor 2 (FLK‐1), scale bar = 50 µm, and 25 µm (insets). The data presented were representative or average of three independent experiments. Abbreviations: DAPI, 4′,6‐diamidino‐2‐phenylindole; ECs, endothelial cells; iPS, induced pluripotent stem; LNA‐199b, locked nucleic acid inhibitor of miR‐199b; LNA‐Ctrl, a negative control; Pre‐199b, a synthetic precursor for miR‐199b; Pre‐ctrl, the nontargeting control.

In this study, mouse iPS cells were generated and differentiated into EC‐like cells as described previously 21. In brief, undifferentiated mouse iPS cells (Fig. 1A, left panel) were subjected to differentiation by seeding the cells on type IV mouse collagen‐coated dishes in DM supplemented with 50 ng/ml VEGF from 0 to 8 days. The differentiated cells acquired a typical morphology of ECs (Fig. 1A, middle panel) and started expressing EC markers such as CD144 and CD31 in both protein (Fig. 1A, right panel), and mRNA levels (Fig. 1B). During EC differentiation, the expression levels of miR‐199b were tested and found to be upregulated in a time‐dependent manner (0–8 days). Notably, enhanced expression of miR‐199b was detected in the late stages of EC differentiation (day 8) (Fig. 1C). To elucidate the regulatory role of miR‐199b in EC differentiation, a synthetic precursor for miR‐199b (Pre‐199b) was used to overexpress miR199b. iPS cells were forced to differentiate toward ECs for 4 days and then efficiently transfected with either precursor molecules (pre‐199b) or the chemically synthesized miR‐199b inhibitor (LNA‐199b). The cells were harvested 48–72 hours later, and subjected to further analysis. The results revealed a significant upregulation and diminution of miR‐199b expression, respectively (Fig. 1D). The Pre‐199b induced enhanced expression of the EC markers CD144 and CD31 in both protein and mRNA levels (Fig. 1E, 1F, and Supporting Information Fig. S1). These results were also confirmed by FACS analysis (Fig. 1G–1H) and real‐time PCR (Fig. 1I). Conversely, knockdown of miR‐199b by LNA‐199b significantly decreased the EC marker expression in both protein (Fig. 1E, 1F, and Supporting Information Fig. S1), and mRNA levels (Fig. 1I). Importantly enhanced Pre‐199b expression resulted in a concomitant rise in VEGF receptor 2 (FLK‐1) as immunofluorescence staining images show in Figure 1J. These initial observations reveal an important role of miR‐199b in EC differentiation derived from iPS cells.

### MiR‐199b Modulates VEGF Transcriptional Activation and Secretion

**Figure 2:**
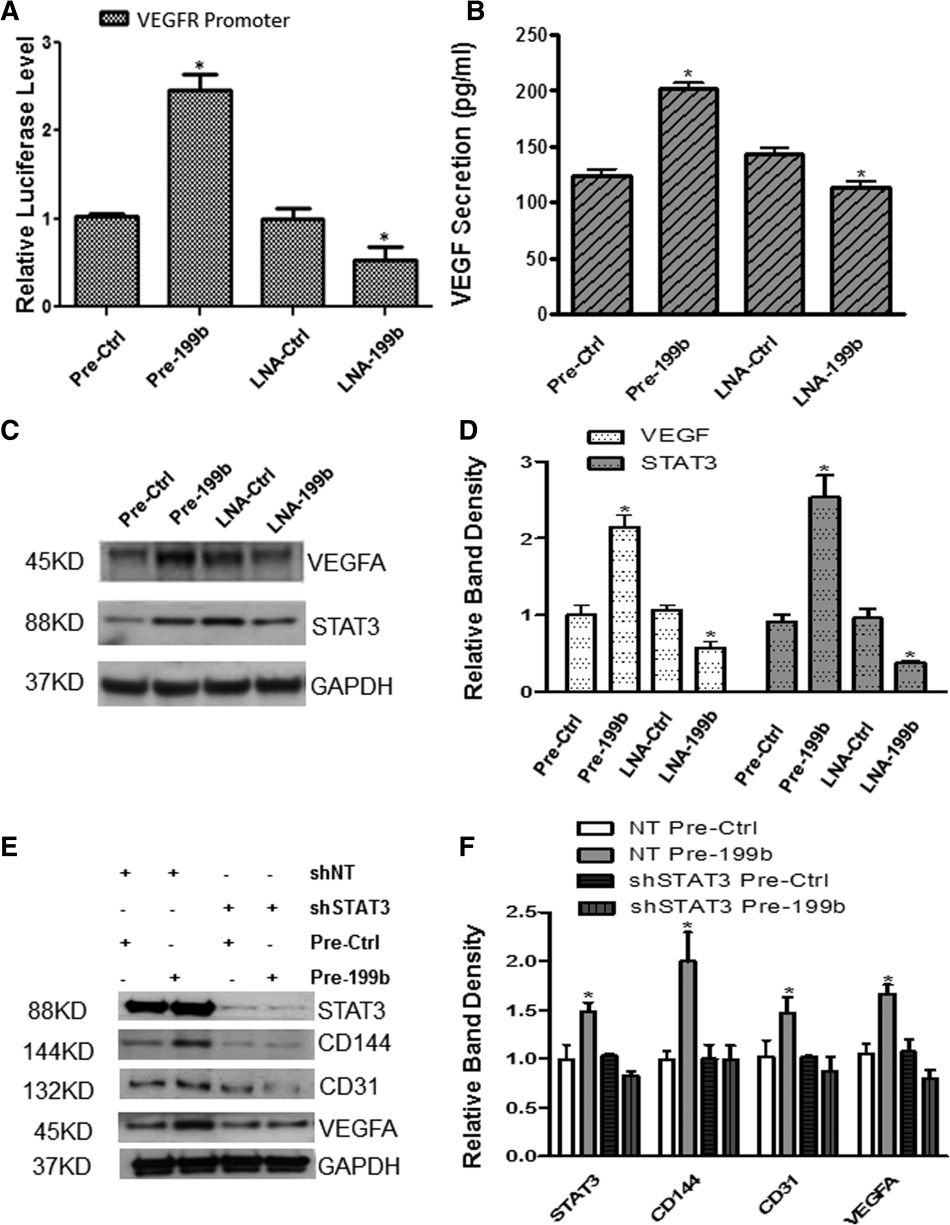
MiR‐199b modulates VEGF transcriptional activation and secretion though STAT3. **(A):** Induced pluripotent stem (iPS) cells were forced to differentiate toward endothelial cells (ECs) for 4 days by seeding the cells on Collagen IV and culture with differentiation medium in the presence of VEGF and then transfected with either Pre‐199b or LNA‐199b and luciferase reporter plasmid for the VEGF receptor. Forty‐eight hours later, the cells were lysed and luciferase assays showed that Pre‐199b modulates the transcriptional activation of VEGF receptor. **(B):** Enzyme‐linked immunosorbent assays demonstrated that the Pre‐199b is implicated in the secretion of VEGF during EC differentiation from iPS cells on day 6. **(C, D):** Pre‐199b induced the expression of VEGF in both protein and mRNA levels in parallel with the expression levels of STAT3. Conversely, LNA‐199b significantly suppressed the expression of VEGF and STAT3, as Western blots and real‐time PCRs showed, respectively. **(E):** iPS cells were forced to differentiate toward ECs, and when STAT3 was knocked down by shRNA, Pre‐199b was unable to induce the expression of VEGF and subsequently the EC marker expression CD144 and CD31 and (**F**, quantification) (data are means ± SEM [*n* = 3], *, *p* < .05). The data presented were representative or average of three independent experiments. Abbreviations: LNA, locked nucleic acid; shNT, short hairpin RNA nontargeting; shSTAT3, short hairpin RNA STAT3; VEGF, vascular endothelial growth factor.

VEGF is a critical regulator of EC differentiation and vasculogenesis during development. Additional experiments have been performed to investigate the potential role of miR199b in VEGF transcriptional activation and secretion. Mouse iPS cells were differentiated toward ECs in the presence of VEGF for 4 days, and then transfected with Pre‐199b or Pre‐Ctrl, and LNA‐199b or LNA‐Ctrl. Forty‐eight, and seventy‐two hours later, the cells were subjected to luciferase assays, and supernatants were collected and ELISA assays were performed. The results have revealed that Pre‐199b modulated the transcriptional activation of VEGF receptor as determined by luciferase assays (Fig. 2A). Furthermore, induced expression or suppression of miR‐199b significantly enhanced or impaired transcriptional activation and secretion of VEGF (Fig. 2A, 2B). Among the transcription factors essential to VEGF gene transcription are the hypoxia‐inducible factor‐1, Sp1, and STAT3 which share homology including consensus sites for Sp1/Sp3, AP2, Egr1, STAT3, and HIF1. It was demonstrated that Pre‐199b induced the expression of VEGF in both protein and mRNA levels in parallel with the expression levels of STAT3 (Fig. 2C, 2D). Conversely, in the presence of the LNA‐199b, both VEGF and STAT3 expression were significantly reduced in both protein and mRNA levels (Fig. 2C, 2D). In order to investigate whether miR‐199b regulated the VEGF expression through the activation of the transcription factor STAT3, Pre‐199b was overexpressed during EC differentiation. When STAT3 was knocked down using shRNA, Pre‐199b was unable to induce expression of VEGF or EC markers (Fig. 2E, 2F). These findings highlight the regulatory role of miR‐199b in VEGF expression which is mediated, at least in part, through transcriptional activation of STAT3.

### MiR‐199b Targets the Notch Ligand Jagged1

**Figure 3:**
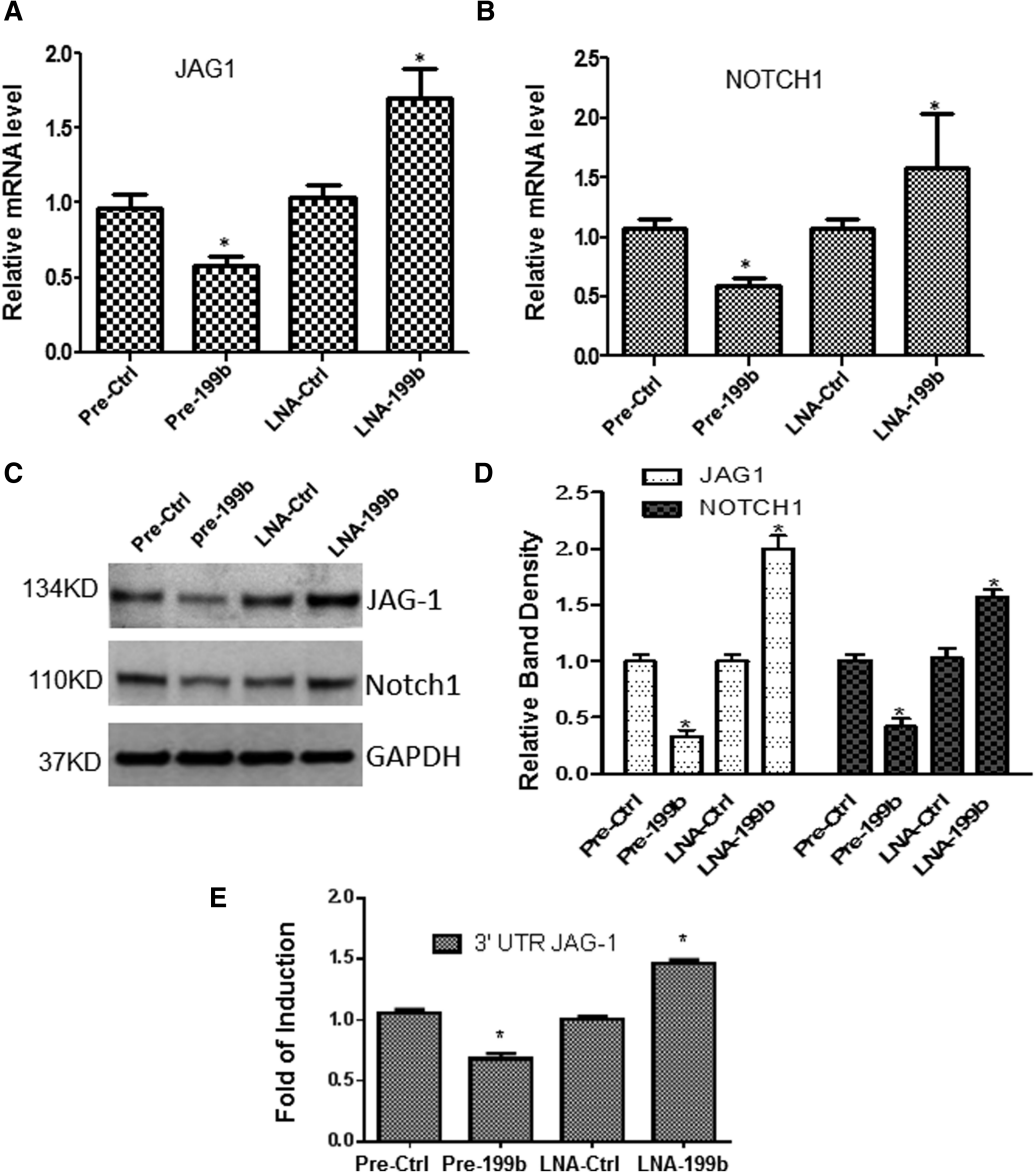
MiR‐199b targets the Notch ligand Jagged1. Pre‐199b suppressed the expression of Notch ligand Jagged1 (JAG1) **(A)** and subsequently the Notch 1 **(B)** in mRNA and protein levels (**C**, and quantification in **D**) during endothelial cell (EC) differentiation from induced pluripotent stem (iPS) cells (data are means ± SEM [*n* = 3], *, *p* < .05). LNA‐199b induced the expression of both JAG1 and Notch (A–D). **(E):** iPS cells were forced to differentiate toward ECs for 4 days, and cotransfections of Pre‐199b or LNA‐199b with the luciferase plasmid of the 3′UTR‐JAG1 were performed. Forty‐eight hours later, the cells have subjected to luciferase analysis demonstrating that the 3′UTR of the JAG1 is a direct target of miR‐199b (data are means ± SEM [*n* = 3], *, *p* < .05). The data presented were representative or average of three independent experiments. Abbreviations: LNA, locked nucleic acid; 3′UTR, three prime untranslated region.

The Notch pathway is a highly conserved signaling system that controls a diversity of growth, differentiation, and patterning processes. In growing blood vessels, sprouting of endothelial tip cells is inhibited by Notch signaling and the precise equilibrium between two Notch ligands with distinct spatial expression patterns and opposing functional roles regulates angiogenesis. Interestingly, further experiments have demonstrated that the Pre‐199b suppressed the expression of JAG1 and Notch 1 while the LNA‐199b induced the expression of both JAG1 and Notch 1 (Fig. 3A–3D). In addition, luciferase assays revealed that the 3′UTR of the JAG1 is a direct target of Pre‐199b during EC differentiation from iPS cells (Fig. 3E). These findings suggest that miR‐199b targets the Notch ligand JAG1 during differentiation toward ECs derived from iPS cells.

### MiR‐199b Modulates STAT3‐VEGF Activation Through Targeting JAG1

**Figure 4:**
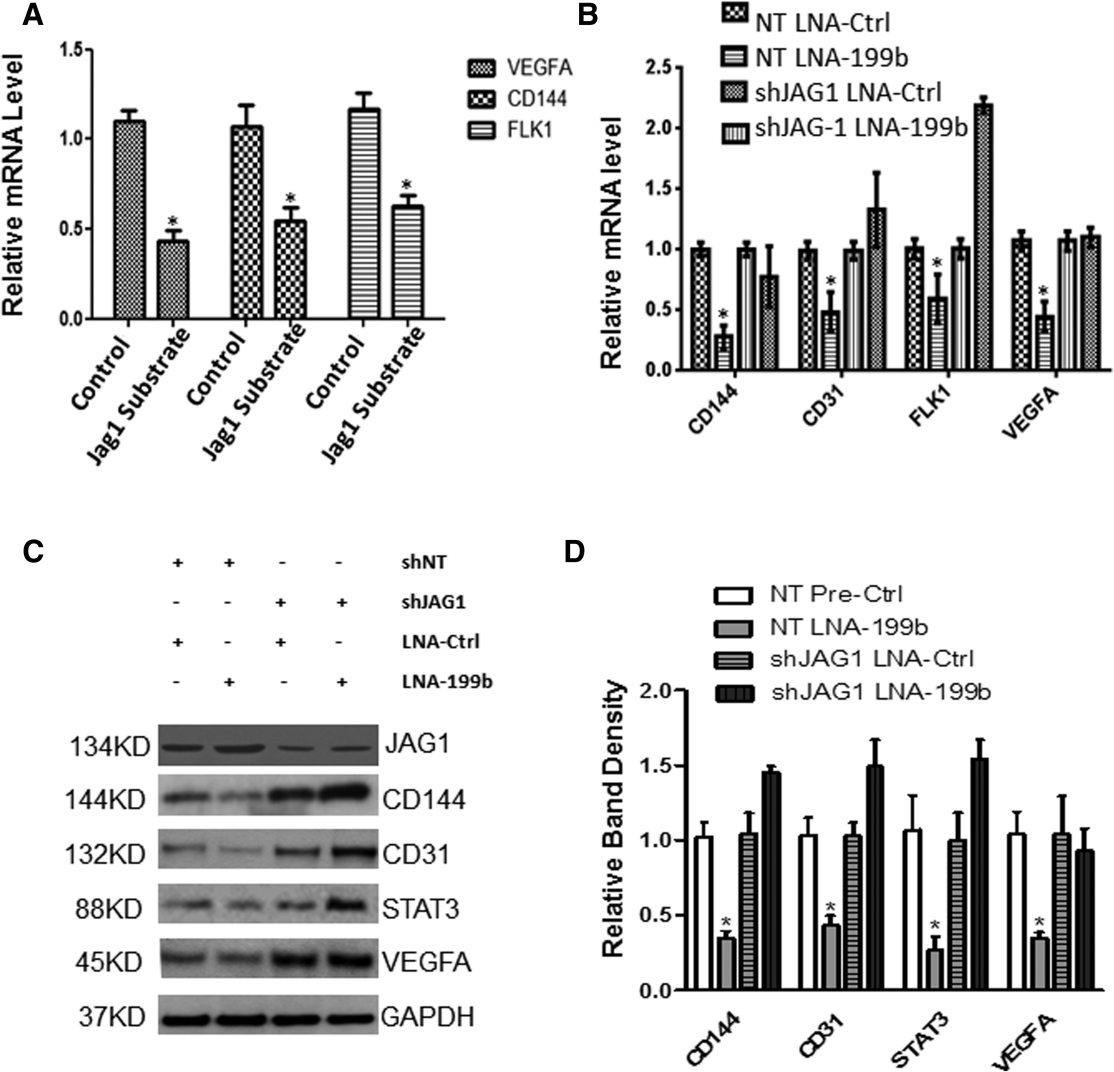
MiR‐199b modulates STAT3‐VEGF activation through targeting JAG1. Mouse induced pluripotent stem (iPS) cells were seeded on 5 µg/ml Jag‐1‐coated plates and cultured in differentiation medium (DM) supplemented with vascular endothelial growth factor (VEGF) for 6 days. Then the cells have been harvested and subjected to further analysis and the expression of endothelial cell (EC) markers were detected by real‐time PCR. **(A):** The differentiated cells in Jag1‐mediated substrate significantly reduced the expression of EC marker CD144, the expression of VEGF and VEGF receptor (FLK‐1), when compared with the control experiments (cells seeded on collagen IV‐coated dishes) (data are means ± SEM [*n* = 3], *, *p* < .05) at day 6 during EC differentiation. Control: iPS cells seeded on collagen IV‐coated dishes, Jag1 Substrate: the recombinant rat Jagged 1 FC Chimera (Product Code: 599‐JG100). **(B):** iPS cells were forced to differentiate toward ECs on collagen IV‐coated dishes in DM media supplemented with VEGF and the JAG1 was knocked down by shRNA. Then, the inhibitory effect of the LNA‐199b inhibitor in EC markers and VEGF and VEGFR (FLK‐1) was ablated in both mRNA (B) and protein levels **(C)**, and quantification in **(D)**. Knockdown of JAG1 by shRNA during EC differentiation showed a robust induction of STAT3 and VEGF in the presence of the miR‐199b inhibitor in protein level as displayed by Western blots (C, D) (data are means ± SEM [*n* = 3], *, *p* < .05). The data presented were representative or average of three independent experiments. Abbreviations: LNA, locked nucleic acid; shJAG1, short hairpin RNA JAG1; shNT, short hairpin RNA nontargeting.

In order to investigate whether JAG‐1 is involved in EC differentiation, mouse iPS cells were seeded on JAG1 substrate and cultured in DM media supplemented with VEGF to induce EC differentiation. It was revealed that the JAG1‐mediated substrate significantly reduced the expression of CD144, VEGF, and the VEGF receptor (FLK‐1) during EC differentiation (Fig. 4A). Importantly, when JAG1 was knocked down by shRNA, the inhibitory effect of LNA‐199b was ablated (Fig. 4B, 4C). Notably, knockdown of JAG1 resulted in the robust induction of STAT3 and VEGF in the presence of LNA‐199b (Fig. 4C, 4D). These results suggest that miR‐199b modulates STAT3‐VEGF activation through targeting JAG1.

### MiR‐199b May Act as a Phenotypic Switch in Vascular Cell Differentiation

**Figure 5:**
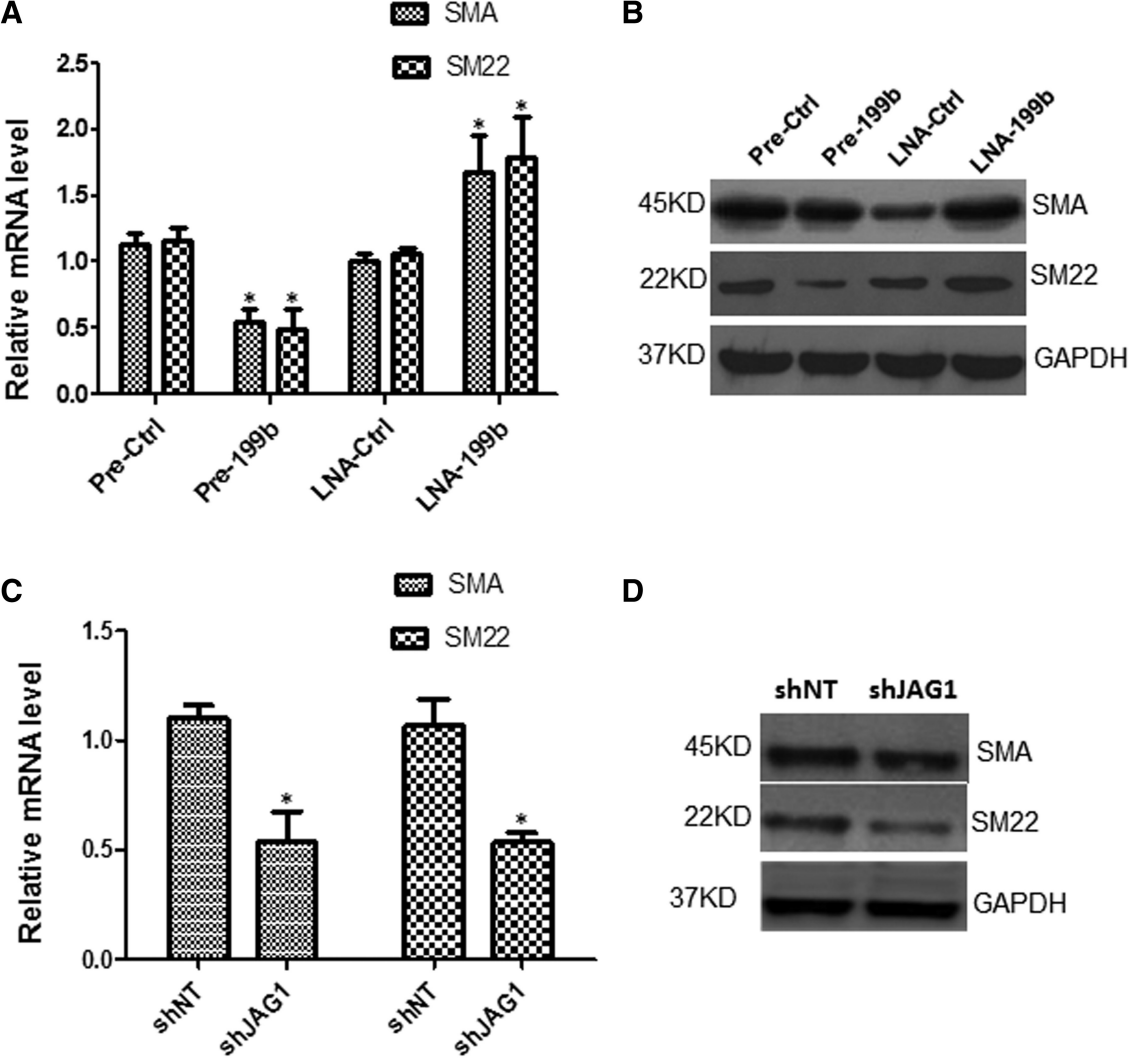
MiR‐199b may have a role as a phenotypic switch in vascular cell differentiation. Induced pluripotent stem (iPS) cells were seeded on collagen IV‐coated dishes and cultured with differentiated media in the absence of vascular endothelial growth factor for 4 days and forced to differentiate toward vascular progenitor cells. (A) Overexpression of Pre‐199b suppressed the expression of smooth muscle markers (SMCs) such as SMA and SM22 in mRNA **(A)** and protein levels **(B)**, as real‐time and Western blots showed, respectively, on day 6 during the vascular cell differentiation. LNA‐199b favored the induction of the SMC markers (A, B). **(C):** When iPS cells were forced to differentiate toward vascular progenitor cells and JAG‐1 was knocked down by shRNA on day 4, a significant suppression of SMCs markers such as SMA and SM22 in mRNA and protein levels is shown in panels (C) and **(D)** (data are means ± SEM [*n* = 3], *, *p* < .05), on day 6 of differentiation. The data presented were representative or average of three independent experiments. Abbreviations: LNA, locked nucleic acid; shJAG1, short hairpin RNA JAG1; shNT, short hairpin RNA nontargeting.

In parallel with the EC experiments, Pre‐199b also served in suppressing the expression of VSMC markers SMA and SM22 during differentiation from iPS cells (Fig. 5A, 5B), when iPS cells were seeded on Collagen IV and cultured in DM media in the absence of VEGF. Conversely, the LNA‐199b favored the induction of the SMC markers (Fig. 5A, 5B). Further support to this notion has been given by the fact that knockdown of JAG1 by shRNA during differentiation toward vascular progenitor cells, resulted in suppression of SMCs markers in mRNA and protein levels in Figure 5C, 5D, respectively. These results provide a link between miR‐199b, the inhibition of JAG1 and SMC differentiation. Based on the fact that both EC and VSMCs are derived from a common vascular progenitor, additional experiments were performed in the presence of VEGF. Mouse iPS cells were subjected to vascular cells differentiation in DM media supplemented with VEGF and on day 4, the cells were transfected with Pre‐199b or Pre‐ Ctrl. Forty‐eight hours later, the cells were harvested and FACS analysis was performed. The results have revealed that Pre‐199b induced the expression of EC markers as shown in Figure 1G, 1H, whilst in parallel resulted in suppression of the VSMC marker SMA (Supporting Information Fig. S2A, S2B). These findings could suggest that miR‐199b might have a potential role to act as a phenotypic switch during vascular cell differentiation.

### MiR‐199b Induced Angiogenesis In Vitro and In Vivo

**Figure 6:**
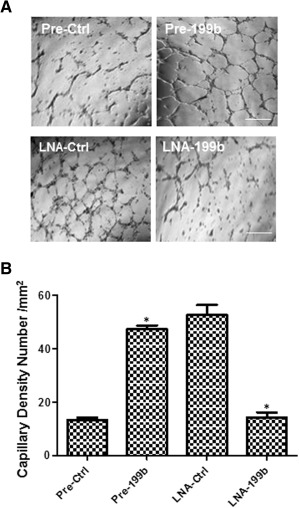
MiR‐199b induced angiogenesis in vitro. Induced pluripotent stem cells were seeded on collagen IV‐coated plates and cultured in differentiation medium in the absence of vascular endothelial growth factor (VEGF) for 4 days, then the Pre‐199b or Pre‐Ctrl was introduced to the cells by transfection (in the absence of VEGF). Forty‐eight hours later, the cells have been subjected to Matrigel plug assays in vitro. **(A):** It is shown that Pre‐199b formed vascular structures within few hours in in vitro Matrigel assays in comparison to the control where less defined vascular structures were observed (A, top panel, and quantification in **B**) (scale bar = 50 µm). Additional experiments were performed in the presence of the VEGF to induce endothelial cell differentiation first and then LNA‐199b was introduced by transfection, showing that the LNA‐199b suppressed the formation of vascular structures in in vitro Matrigel plugs assays in comparison to the control cells (A, lower panel, and quantification in B) (scale bar = 50 µm) (data are means ± SEM [*n* = 3], *, *p* < .05). The data presented were representative or average of three independent experiments. Abbreviation: LNA, locked nucleic acid.

**Figure 7:**
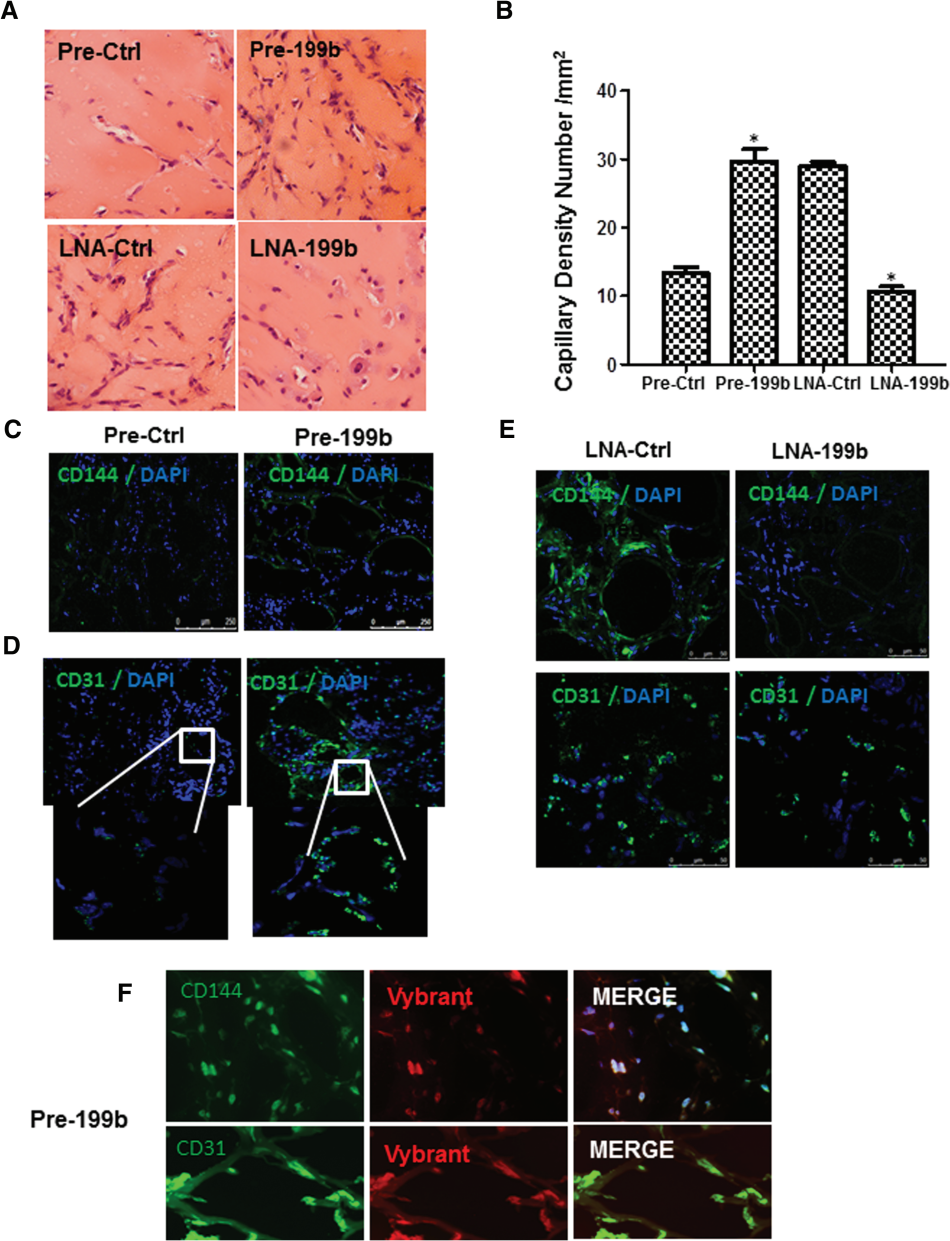
Modulation of MiR‐199b expression induces angiogenesis and expression of endothelial cell (EC) markers in vivo. In a similar way as in Figure 6, the cells were subjected to Matrigel plug assays in vivo. **(A):** Mouse induced pluripotent stem (iPS) cells were seeded on collagen IV‐coated plates and cultured in differentiated media in the absence of vascular endothelial growth factor (VEGF) for 4 days and the Pre‐199b or Pre‐Ctrl was introduced to the cells by transfection. Forty‐eight hours later, the cells were labeled with Vybrant and subjected to Matrigel plug assays in vivo. It is shown that Pre‐199b induced the formation of well‐defined vascular structures within 7 days in in vivo Matrigel assays in comparison to the control where less well‐formed vascular structures were observed (A, top panel [scale bar = 50 µm], and quantification in **B**). Mouse iPS cells were seeded on collagen IV‐coated plates and cultured in differentiated media in the presence of VEGF for 4 days to induced EC differentiation first and LNA‐199b or LNA‐Ctrl was introduced to the cells by transfection. Forty‐eight hours later, the cells were labeled with Vybrant and subjected to Matrigel plug assays in vivo. LNA‐199b inhibitor suppressed the formation of vascular structures in in vivo Matrigel plugs assays in comparison to the control cells (A, lower panel [scale bar = 50 µm], and quantification in B) (data are means ± SEM [*n* = 3], *, *p* < .05). **(C, D):** Frozen sections were stained with EC‐specific markers demonstrating that over‐expression of Pre‐199b resulted in well‐formed vascular structures and enhanced engraftment ability compared to the control cells as staining for CD144 and CD31 showed (scale bar for C; 250 µm, for D; 50 µm and insets in D; 25 µm). DAPI was used and stained the cell nucleus. **(E):** MiR‐199b inhibition by LNA‐199b resulting in a reduced vascular formation and engraftment ability as it is revealed by staining with EC markers, scale bar = 50 µm. Importantly, the Pre‐199b overexpressing cells were positively costained for the EC markers CD31 and CD144 and Vybrant, revealing that the injected mouse iPS cells formed the vascular structures in matrigel plugs in vivo **(F)**, scale bar = 50 µm (upper panel), 25 µm (lower panel). The data presented were representative or average of three independent experiments. Abbreviations: DAPI, 4′,6‐diamidino‐2‐phenylindole; LNA, locked nucleic acid.

To evaluate the role of miR‐199b in angiogenesis, miR‐199b was altered by overexpression using Pre199b or inhibition using LNA‐199b. Since previous results indicated that miR‐199b induced the secretion of VEGF, we decided to perform the overexpression experiments with Pre‐199b in the absence of exogenous VEGF using Matrigel plug assays in vitro and in vivo. In brief, mouse iPS cells were seeded on collagen IV‐coated plates and cultured in DM media in the absence of VEGF for 4 days and the Pre‐199b mimic or Pre‐Ctrl were introduced to the cells by transfection. Forty‐eight hours later, the cells were labeled with Vybrant and subjected to Matrigel assays plugs in vitro and in vivo. Differentiated cells in the presence of a Pre‐199b showed significantly enhanced vascular tube formation in vitro (Fig. 6A, and quantification in 6B) and vessel formation in vivo (Fig. 7A, and quantification in Fig. 7B) when compared with Pre‐Ctrl. It is important to highlight that these experiments using Pre‐199b or Pre‐Ctrl have been performed in the absence of VEGF and that the enhancement of vascular tube formation in both in vitro and in vivo results from the secretion of VEGF by Pre‐199b. In order to test whether inhibition of miR199b by LNA‐199b could result in suppression of vascular tube formation in vitro and in vivo, further experiments were performed in the presence of VEGF to induce EC differentiation first following inhibition by LNA‐199b. It was demonstrated that LNA‐199b suppressed the formation of vascular structures in both in vitro and in vivo assays in comparison to the LNA‐Ctrl cells (Figs. 6A, 7A, and quantification in Figs. 6B, 7B). The Matrigel plugs were harvested from mice 7 days post‐transplantation, and subjecting to immunohistochemistry revealing the presence of CD31 and CD144 EC markers (Fig. 7C–7E). ECs over‐expressing Pre‐199b displayed more well‐formed vascular structures and enhanced engraftment ability when compared with the control cells (Fig. 7C, 7D). In contrast, differentiated ECs transfected with LNA‐199b displayed reduced vascular formation and engraftment ability (Fig. 7E). Importantly, the Pre‐199b overexpressing cells were costained positive for the EC markers CD31 and CD144, as well as Vybrant, revealing that the injected mouse iPS cells formed the vascular structures in matrigel plugs in vivo (Fig. 7F).

## Discussion

iPS cells have the potential to differentiate into any adult cell and they have already significantly impacted the study of disease and the discovery of potential stem cell‐based therapies. Ultimately, this technology may represent the most attractive cellular approach for regenerative medicine 1, 2, 3. In this study, we report that miR‐199b is involved in iPS cell differentiation to ECs. MiR‐199b is upregulated during EC differentiation, and significantly enhanced expression of miR‐199b occurs in the late stages of this process. Notably, miR‐199b targets the Notch ligand JAG1, which results in VEGF transcriptional activation and secretion through the transcription factor STAT3. Our findings provide the first evidence that miR‐199b regulated Notch signaling is crucial for VEGF production, which directs iPS cell differentiation toward endothelial lineages.

MiR‐199b has been described as a potential Notch regulator through targeting the transcription factor HES1 12, 26. Notch signaling is essential for vascular development, homeostasis, and angiogenesis although the molecular basis for its upstream regulation remains poorly understood. It has been previously reported that the Notch ligand Delta‐like 4 negatively regulates endothelial tip cell formation and vessel branching 27. In this study, miR‐199b was shown to induce the secretion and transcriptional activation of VEGF during EC differentiation from iPS cells. VEGF is the earliest marker for the endothelial lineage during development 28, 29. To shed light on the underlying mechanisms, it has been revealed that the activation of VEGF by Pre‐199b is mediated, at least in part, by STAT3 activation. Interestingly, knockdown of STAT3 by shRNA ablated secretion of VEGF and the expression of EC markers mediated by Pre‐199b. It is established that constitutive STAT3 activity upregulates VEGF 30, as STAT3 protein binds to the VEGF promoter inducing VEGF promoter activity 30. Therefore, our data indicate that inhibition of JAG‐1 by Pre‐199b leads to activation of STAT3 and induction of EC differentiation from iPS cells. In parallel, we found that inhibition of JAG‐1 by Pre‐199b leads to the suppression of SMCs differentiation. In addition, inhibition by LNA‐199b resulted in robust induction of SMC marker expression. Ferreira et al. in 2007 have reported that vascular progenitor cells isolated from human embryonic stem cells gave rise to EC and SMC‐like cells and formed vascular networks in vivo 6, providing strong evidence that ECs and SMCs were derived from a common progenitor. Therefore, it is possible that a molecular switch could regulate the vascular cell fate and thereby maintain the balance of these two cell lineages during development, disease‐repairing process, and cell differentiation. This notion is in agreement with a recent study showing that TGF‐beta induced JAG1 and SMC markers, in an independent manner of the activation of SMAD3 and Rho kinase. In the same study, it was also shown that knocking down JAG1 completely blocked MYOCD expression, suggesting that this ligand plays an important role in TGF‐beta‐induced expression of SMC markers. Notch activation in human embryonic stem cells suppresses appearance of EC markers 31. Our findings may suggest a potential unique role of miR‐199b as a regulator of Notch signaling. This may imply a vascular cell phenotypic switch, possibly suggesting previously unrecognized mechanisms involved in iPS cells differentiation.

This study highlights the potential of Pre‐199b in regulating angiogenesis in both in vitro and in vitro Matrigel plug assays, even in the absence of exogenous VEGF. In previous studies, iPS cell‐derived ECs incorporated into the regenerating microvasculature of ischemic tissue, thereby enhancing perfusion and improving function 32, 33. Currently, the potential of human iPS cells to differentiate toward therapeutic cells is based on directed empiricism. They are also entirely dependent on combinations of growth factors, media, and matrices to favor the desired lineage. In regards to vascular regeneration, it is important to understand regulatory pathways such as epigenetic alterations, transcriptional activity, and microRNA patterns associated with the differentiation processes. Only then, refined experimental protocols may reproducibly guide human iPS cells to a vascular lineage 34, 35 and enable clinical application 36, 37, 38. In this regard, the role of miR‐199b during human iPS cells differentiation toward ECs has been evaluated and preliminary data showed that Pre‐199b induced further EC marker expression during EC differentiation derived from human iPS cells. In contract, LNA‐199b resulted in the inhibition of EC differentiation (Supporting Information Fig. S3). These results confirm the potential role of miR‐199b in EC differentiation derived from human iPS cells as well.

In summary, our data strongly support the notion that miR‐199b modulates VEGF transcriptional activation and secretion through STAT3 and JAG1. Notably, further elucidation of the underlying mechanisms has revealed that through targeting of JAG1, miR‐199b inhibited the differentiation toward SMCs (Supporting Information Fig. S4). This points toward a novel role for miR‐199b as a potential regulator of the phenotypic switch during vascular cell differentiation. Finally, the effect of miR‐199b to secrete VEGF and induce angiogenesis has been evaluated in in vitro and in vivo Matrigel plug assays. This study provides support to the notion that with diligent efforts to understand the molecular mechanisms of vascular cell fate, stem cell regenerative therapy may ultimately become a reality and shift the paradigm to treat cardiovascular disease.

## Conclusions

This study provides support to the notion that with diligent efforts to understand the molecular mechanisms of vascular cell fate, stem cell regenerative therapy may ultimately become a reality and shift the paradigm to treat cardiovascular disease.

## Author Contributions

T.C.: collection and/or assembly of data, data analysis and interpretation, and final approval of manuscript; A.M.: conception and design, collection and/or assembly of data, data analysis and interpretation, provided of study materials, manuscript writing, and final approval of manuscript; S.K.: collection and/or assembly of data and manuscript writing; A.C.: collection and/or assembly of data; S.T.G.: provided of study materials and data analysis and interpretation; Y.H.: collection and/or assembly of data and data analysis and interpretation; A.W.S.: provided of study materials and manuscript writing; L.Z.: financial support and final approval of manuscript; Q.X.: conception and design, financial support, data analysis and interpretation, provided of study materials, manuscript writing, and final approval of manuscript. T.C. and A.M. contributed equally to this article.

## Disclosure of Potential Conflicts of Interest

The authors indicate no potential conflicts of interest.

## Supporting information

Supplementary Methods

Supplementary Figure S1

Supplementary Figure S2

Supplementary Figure S3

Supplementary Figure S4
