# Supplementary material for: MicroRNA‐199b Modulates Vascular Cell Fate During iPS Cell Differentiation by Targeting the Notch Ligand Jagged1 and Enhancing VEGF Signaling
Source: Stem Cells. 2015 Apr 23;33(5):1405–18. doi: 10.1002/stem.1930 (PMC4737258; doi:10.1002/stem.1930)
Supplement: Supplementary file 1 — Supplementary Methods [file STEM-33-1405-s001.docx]

**Supplementary Methods S1**

**Reverse transcriptase-polymerase chain reaction (RT-PCR)**

RT-PCR and Real Time PCR were performed as described previously[^1^](#_ENREF_1). Total RNA was extracted using the RNeasy Mini Kit (Qiagen) according to the manufacturer's protocol. 2 µg RNA were reversely transcribed into cDNA with random primers by MMLV reverse transcriptase (RT) (Promega). 20-50ng cDNA (relative to RNA amount) were amplified by quantitative RT-PCR.

**Quantitative RT-PCR**

Relative gene expression was determined by Quantitative Real Time PCR, using 2 ng of cDNA (relative to RNA amount) for each sample with the SYBR Green Master Mix in a 20-μl reaction. *Ct* values were measured using the ABI Prism 7000 sequence detector (Applied Biosystems). The 18 S ribosomal RNA served as the endogenous control to normalize the amounts of RNA in each sample. For each sample, PCR was performed in duplicate in a 96-well reaction plate (Eppendorf, twin.tec real time PCR plates). The gene was considered undetectable beyond 35 cycles. The primer sets used for this study are as follows: 18 S, forward, 5′-CCAGTAAGTGCGGGTCATAA-3′ and reverse, 5′-CCGAGGGCCTCA CTAAACC-3′; VE-cadherin, forward, 5′-AAGAAACCGCTGATCGGCA-3′ and reverse, 5′-TCGGAAGAATTGGCCTCTGTC-3′; CD31, forward, 5′-CAAACAGAAACCCGTGGAGA T-3′ and reverse, 5′-ACCGTAATGGCTGTTGGCTTC-3′; Flk-1, forward, 5′-TGAAATTGA GCTATCTGCCGG-3′ and reverse, 5′-TTTGAAGGTGGAGAGTGCCAG-3′; VegfA, forward, 5'-TCACCAAAGCCAGCACATAGGAGA-3', and reverse, 5'-TTACACGTCTG CGGATCTTGGACA-3'; Stat3, forward 5'-AGTCACATGCCACGTTGGTGTTTC-3', and reverse, SMA, Forward, 5'-CGGGCAATTTCCATTGGCTTCTCA-3'; 5’-TCCTGACGCTG AAGTATCCGAT-3’, and reverse 5’-GGCCACACGAAGCTCGTTATAG-3’; SM22, forward 5’-GATATGGCAGCAGTGCAGAG-3’; and reverse 5’-AGTTGGCTGTCT GTGAAGTC-3’; Jag-1, forward 5’-CTGTCCCACTGGTTTCTCTG-3’; and reverse, 5’-GTTCTTGCCCTCATAGTCCTC-3’; Notch 1, forward 5’-ATGTCAATGTTCG AGGACCAG-3’, and reverse, 5’-TCACTGTTGCCTGTCTCAAG-3’. Human CD34, forward 5’-GTGTCTACTGCTGGTCTTGG-3’, and reverse, 5’-AGGGTCTTTTGGGA ATAGCTC-3’, Human CD31, forward 5’- TCAGAAGGACAAGGCGATTG-3’, and reverse, 5’- GTTATGTTGACCACGATGCTG-3’, Human VE-Cadherin, forward 5’ AAACACCTCACTTCCCCATC-3’, reverse 5’-ACCTTGCCCACATATTCTCC-3’.

**Immunoblotting**

The method used was similar to that described previously[^1^](#_ENREF_1). Cells were harvested and washed with cold PBS, re-suspended in lysis buffer (25mM Tris-Cl pH 7.5, 120mM NaCl, 1 mM EDTA pH 8.0, 0.5% Triton X100) supplemented with protease inhibitors (Roche). The cells were then lysed by ultra-sonication (twice, 6 seconds each) (Bradson Sonifier150) to obtain whole cell lysate. The protein concentration was determined using the Biorad Protein Assay Reagent. The whole lysate (50μg) was then applied to SDS-PAGE and transferred to a Hybond PVDF membrane (GE Health), followed by standard Western blotting procedure. The bound primary antibodies were detected by the use of horseradish peroxidase (HRP)-conjugated secondary antibody and the ECL detection system (GE Health). The band density was semi-quantified by Adobe Photoshop software.

**Lentiviral particle transduction**

Lentiviral particles were produced using the MISSION shSTAT3, shJAG-1 DNA plasmids (SIGMA) according to protocol provided and previously described[^1^](#_ENREF_1). The shRNA Non-Targeting vector was used as a negative control. Briefly, 293T cells were transfected with the lentiviral vector and the packaging plasmids, pCMV-dR8.2 and pCMV-VSV-G (both obtained from Addgene) using Fugene 6. The supernatant containing the lentivirus was harvested 48h later, filtered, aliquoted and stored at –80°C. p24 antigen ELISA (Zeptometrix) was used to determine the viral titre. The Transducing Unit (TU) was calculated using the conversion factor recommended by the manufacturer (10^4^ physical particles per pg of p24 and 1 transducing unit per 10^3^ physical particles for a VSV-G pseudotyped lentiviral vector), with 1pg of p24 antigen converted to 10 Transducing Units (TU). For lentiviral infection, iPS were differentiated for 3 days and the cells were incubated with shSTAT3, or shJAG-1 or Non Targeting control (1x10^7^TU/ml), (24 hours prior the transfection with mir-199b or inhibitor) in complete medium supplemented with 10μg/ml of Polybrene for 24h. Subsequently, fresh medium was added to the cells and the plates were returned to the incubator and harvested 72h later for further analysis.

**1. Margariti A, Zampetaki A, Xiao Q, Zhou B, Karamariti E, Martin D, Yin X, Mayr M, Li H, Zhang Z, De Falco E, Hu Y, Cockerill G, Xu Q, Zeng L. Histone deacetylase 7 controls endothelial cell growth through modulation of beta-catenin. *Circ Res*. 2010;106:1202-1211**
