# Supplementary figures and images for: MicroRNA‐199b Modulates Vascular Cell Fate During iPS Cell Differentiation by Targeting the Notch Ligand Jagged1 and Enhancing VEGF Signaling
Source: Stem Cells. 2015 Apr 23;33(5):1405–18. doi: 10.1002/stem.1930 (PMC4737258; doi:10.1002/stem.1930)

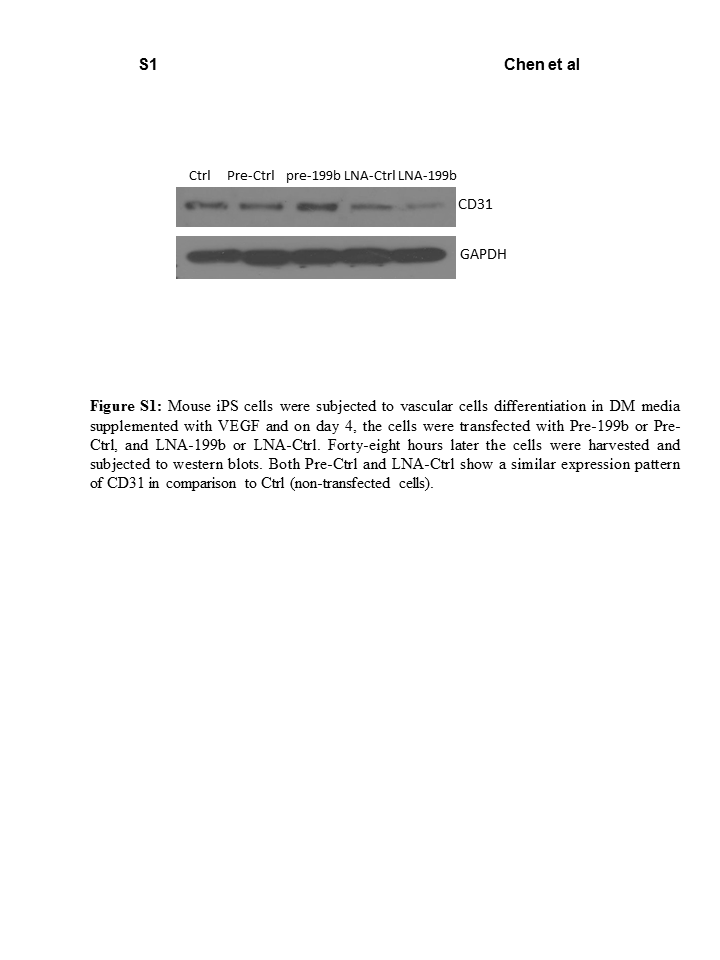

Supplement: Supplementary file 2 — Supplementary Figure S1 [file STEM-33-1405-s002.TIF]

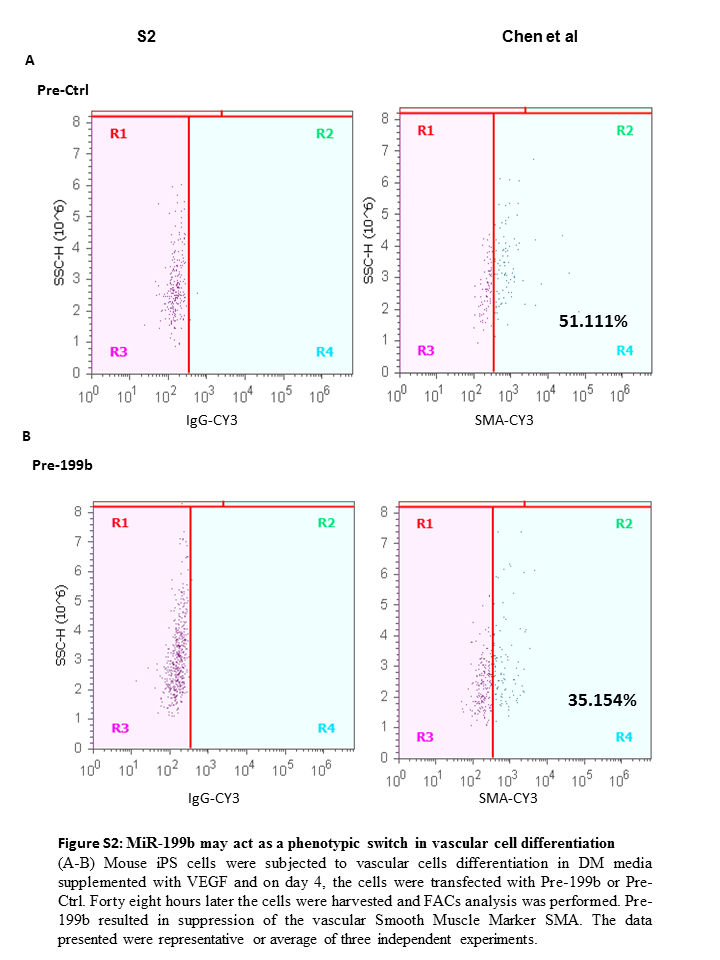

Supplement: Supplementary file 3 — Supplementary Figure S2 [file STEM-33-1405-s003.TIF]

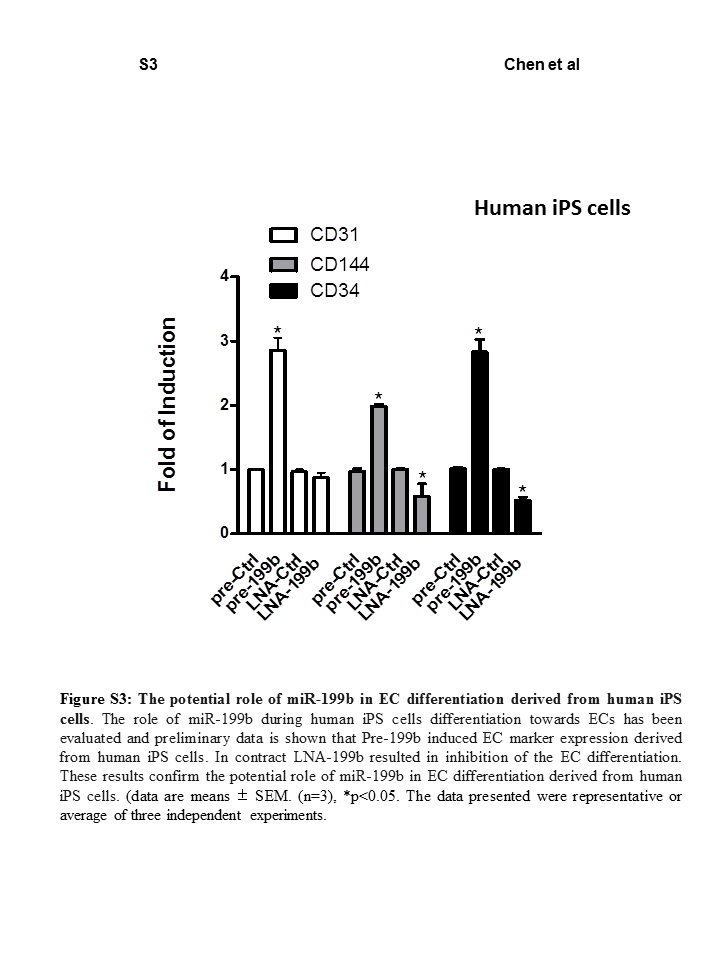

Supplement: Supplementary file 4 — Supplementary Figure S3 [file STEM-33-1405-s004.TIF]

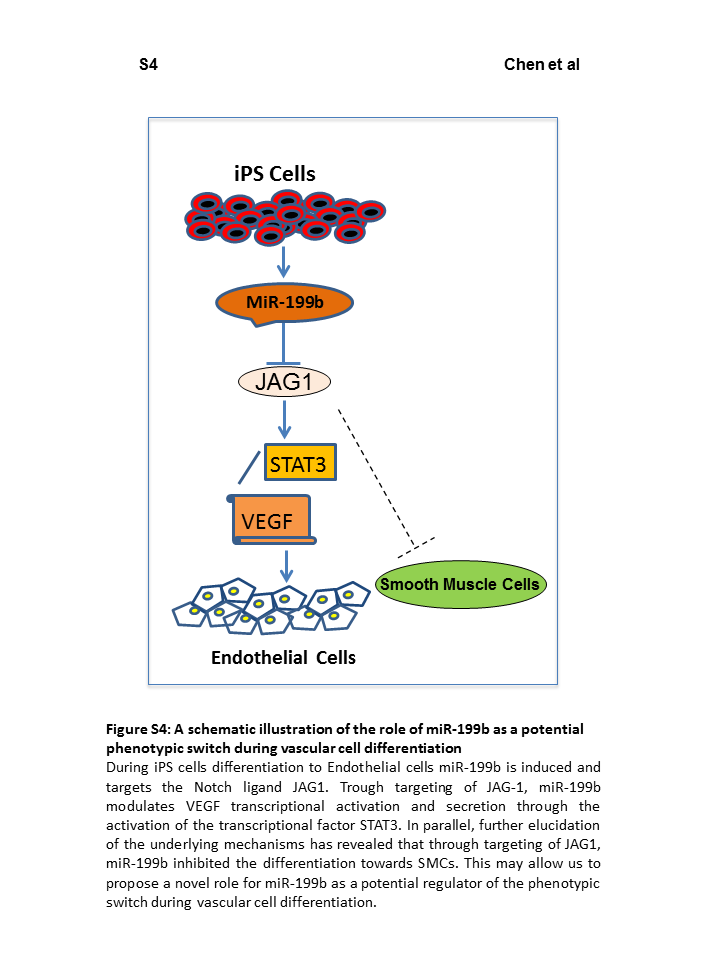

Supplement: Supplementary file 5 — Supplementary Figure S4 [file STEM-33-1405-s005.TIF]
